# Supplementary material for: Chemical Composition and Biological Activity of Five Essential Oils from the Ecuadorian Amazon Rain Forest
Source: Molecules. 2019 Apr 25;24(8):1637. doi: 10.3390/molecules24081637 (PMC6514681; doi:10.3390/molecules24081637)
Supplement: Supplementary file 1 [file molecules-24-01637-s001.pdf]

NMR characterization of isolated germacrene D

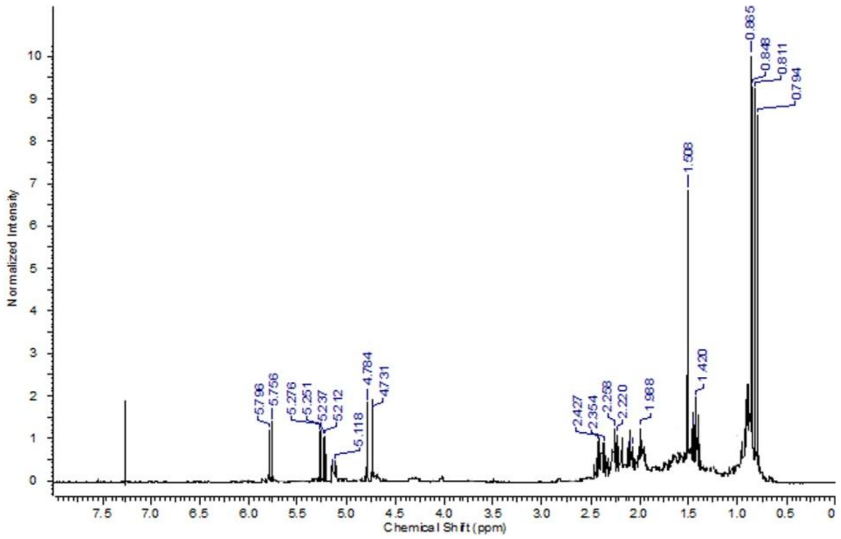

Figure 1S. ^{1}H NMR spectrum of germacrene D, in $CDCl_3$ (recorded with a *Varian Mercury Plus 400*, operating at 400 MHz (^{1}H). The chemical shifts were referenced to the residual solvent signal ($CDCl_3$: 7.26 ppm)

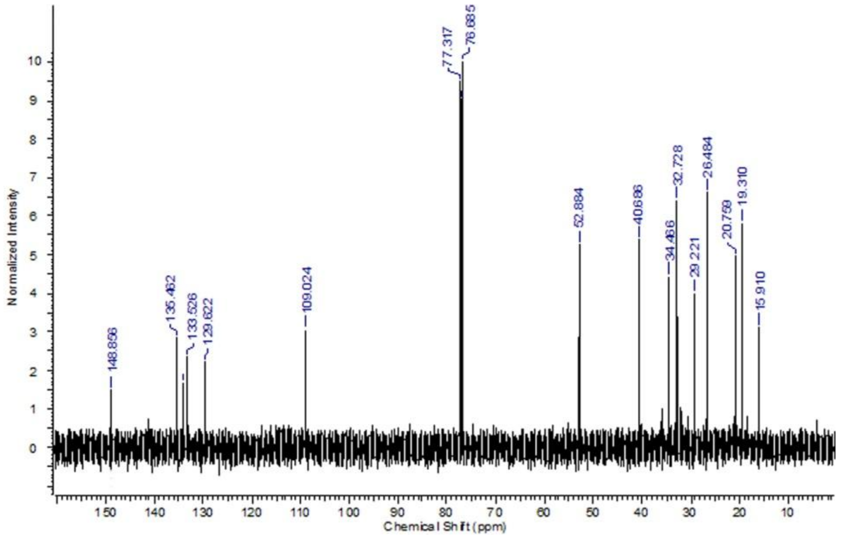

Figure 2S. ^{13}C NMR spectrum of germacrene D, in $CDCl_3$ (recorded with a *Varian Mercury Plus 400*, operating at 100 MHz. The chemical shifts were referenced to the residual solvent signal ($CDCl_3$: 77.16 ppm)
